# Successful Plate Fixation with Long Intramedullary Fibula Bone Graft for Periprosthetic Femur Fracture: A Case Report

**DOI:** 10.3390/medicina58091148

**Published:** 2022-08-24

**Authors:** Kuei-Lin Yeh, Chen-Kun Liaw, Chiou-Shann Fuh, Chu-Song Chen, Chen-Hao Chiang, Kao-Shang Shih

**Affiliations:** 1 Department of Orthopaedics, Ditmanson Medical Foundation Chia-Yi Christian Hospital, No. 539, Zhongxiao Rd., East District, Chia-Yi City 600566, Taiwan; 2 Department of Computer Science and Information Engineering, National Taiwan University, No. 1, Sec. 4, Roosevelt Rd., Da’an District, Taipei City 106216, Taiwan; 3 Department of Orthopaedics, School of Medicine, College of Medicine, Taipei Medical University, No. 250, Wuxing St., Xinyi District, Taipei City 11031, Taiwan; 4 Department of Orthopaedics, Shuang Ho Hospital, Taipei Medical University, No. 291, Zhongzheng Rd., Zhonghe District, New Taipei City 23561, Taiwan; 5 Graduate Institute of Biomedical Optomechatronics, College of Biomedical Engineering, Research Center of Biomedical Device, Taipei Medical University, Taipei City 11031, Taiwan; 6 Department of Orthopaedics, Shin Kong Wu Ho-Su Memorial Hospital, No. 95, Wenchang Road, Shilin District, Taipei City 111045, Taiwan; 7 School of Medicine, Department of Medicine, Fu Jen Catholic University, No. 510, Zhongzheng Rd., Xinzhuang District, New Taipei City 242062, Taiwan

**Keywords:** periprosthetic fracture, osteoporosis, allogenous fibula bone graft, distal femur fracture

## Abstract

*Background and objectives*: Treatment of a displaced or comminuted periprosthetic distal femur fracture is challenging, especially in patients with osteoporosis. In this case report, we shared our successful surgical experience of using a long intramedullary fibula bone graft in a plate fixation surgery for a periprosthetic distal femur fracture in an extremely elderly patient with osteoporosis. *Case report*: A 95-year-old woman with severe osteoporosis (bone mineral density level: −3.0) presented with right knee pain and deformity after a fall, and a right periprosthetic distal femur fracture was identified. The patient underwent an open reduction and an internal plate fixation surgery with the application of a long intramedullary fibular bone graft. Due to a solid fixation, immediate weight-bearing was allowed after the surgery. She could walk independently without any valgus or varus malalignment or shortening 3 months after the surgery. A solid union was achieved 4 months postoperatively. *Conclusions*: We present a case wherein a long intramedullary allogenous fibula strut bone graft was used successfully to treat a right periprosthetic femur fracture in an extremely elderly patient. A long allogenous fibula bone graft can act not only as a firm structure for bridging the bone defect but also as a guide for precise component alignment. We believe this treatment option for periprosthetic fractures is beneficial for achieving biological and mechanical stability and facilitates early mobilization and weight-bearing for the patient.

## 1. Introduction

Total knee arthroplasty (TKA) is one of the most efficacious, successful, and cost-effective treatments for advanced knee arthritis in terms of pain relief, function restoration, and quality-of-life enhancement [1,2,3]. In terms of population expansion, during the first decade of the 21st century, the population of Americans aged 85 to 94 years has increased by nearly 30%, from 3.9 to 5.1 million [4,5]. A substantial increase in the incidence of TKA has been observed [6,7,8]. One complication of TKA is periprosthetic fracture, which occurs because most patients requiring TKA are of advanced age and have osteopenia [9,10]. 

Femoral supracondylar fractures are observed in 0.3–2.5% of all patients with TKA [7,11]. With a continuous increase in the number of TKAs performed, these injuries are expected to become more common [4]. Fracture of the distal femur after TKA is a complex orthopedic problem, particularly in patients with poor bone stock [12]. Patients with TKA are additionally vulnerable to fracture because the presence of the TKA femoral component biomechanically weakens the surrounding supracondylar region of the bone [13]. Thus, the management of femoral periprosthetic fractures is an important issue. 

In most cases, TKA is well fixed and functions well before fracture [14]. Orthopedic surgeons have preferred internal fixation for fractures above well-fixed components [15,16]. Historically, the treatment options for fixation of these fractures included lateral locked plating, retrograde intramedullary nailing, dual plating, or a combination of nailing and plating [17,18]. 

We described the application of a long intramedullary allogenous fibular graft in plate fixation for a periprosthetic distal femur fracture. We hypothesized that a long fibular bone graft would have biomechanics similar to intramedullary nailing fixation. The combination of plating and nailing was considered solid fixation in our patient, allowing for early mobilization and immediate weight-bearing. The patient demonstrated an excellent outcome 4 months before undergoing an open reduction and internal fixation surgery.

## 2. Case Report

**Figure 1:**
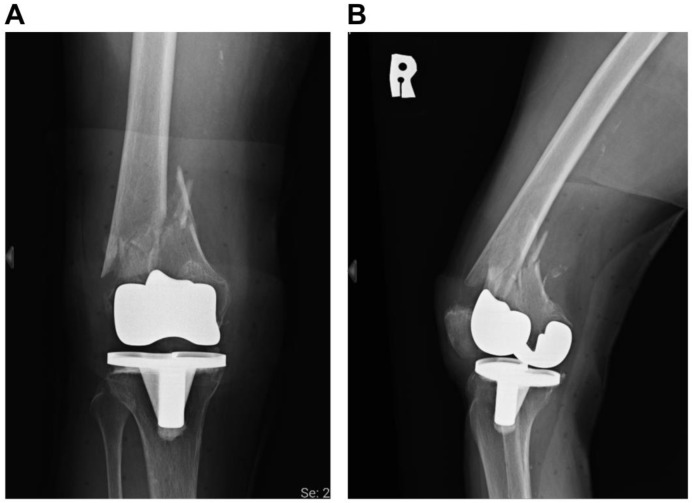
Preoperative radiographic images. (**A**) Anteroposterior (AP) view of knee. (**B**) Lateral view of knee. The patient was diagnosed with a periprosthetic distal femur fracture.

In May 2020, a 95-year-old woman with severe osteoporosis (bone mineral density level: −3.0) presented to our outpatient department with right knee pain and deformity secondary to syncope and a fall episode. Her underlying diseases included hypertension and moderate mitral valve regurgitation. Since she was bothered by her right knee osteoarthritis, she underwent right TKA in 2014. Physical examination revealed pain and crepitus when the patient’s right knee was flexed. No numbness was noted. A plain knee radiograph was subsequently arranged, which led to the diagnosis of a right femur periprosthetic fracture with Su’s classification type II (Figure 1). 

Considering the patient’s advanced age, we discussed the treatment options (including conservative and surgical treatments) with the patient and her family. Before the accident, the patient was able to walk independently indoors, but needed help or supervision while walking outdoors. Subsequently, the patient decided to undergo an open reduction and internal fixation surgery for the periprosthetic fracture of the right femur. 

**Figure 2:**
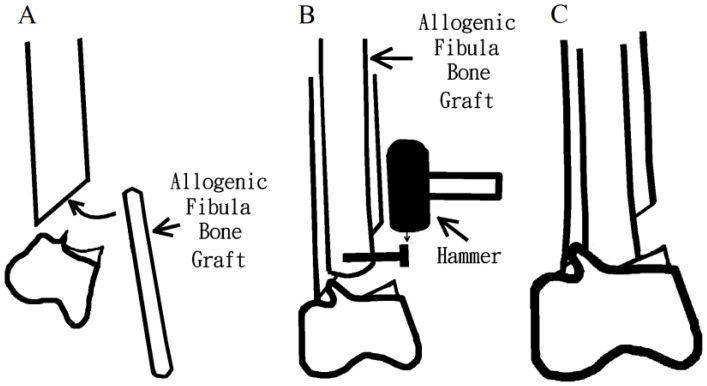
Line drawings depicting the method for insertion of the allograft. (**A**) The fibula bone graft was inserted; (**B**) A temporary screw was inserted at the distal part of the bone graft, and pulled the bone graft to the appropriate position; (**C**) The bone graft placement to the accurate position was accomplished, and prepared for plate fixation.

**Figure 3:**
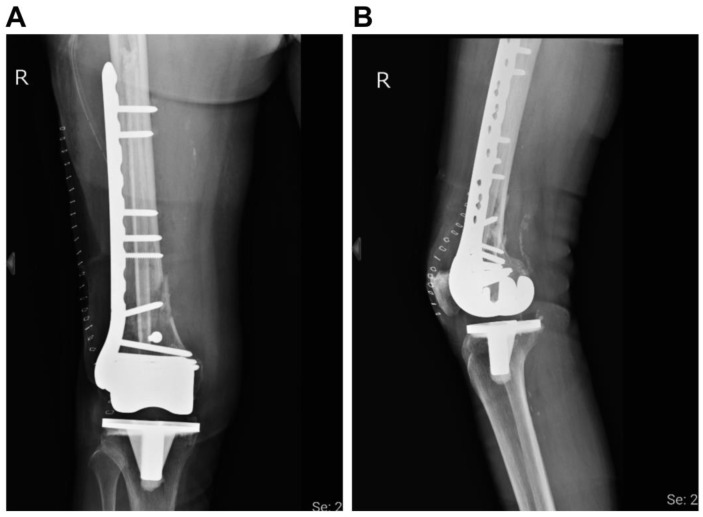
Postoperative radiographic images. (**A**) Anteroposterior (AP) view of knee. (**B**) Lateral view of knee. The periprosthetic distal femur fracture is fixed with a locking plate and an allogenous fibula bone graft.

After completing the preoperative survey, the surgery was performed the day after admission. The patient was placed in the supine position. A lateral approach was used for the surgery. Upon exploring the fracture site, a bone defect was observed on the lateral side. Therefore, a long non-vascularized fibula allograft of approximately 24 cm in length was inserted as the intramedullary structure. This bone allograft was harvested from a deceased donor 10 months prior and was stored in our authorized bone bank. The largest width of the fibular bone graft was 11 mm. We first inserted the fibula bone graft in a retrograde manner. We made sure the bone graft was placed in the bone canal. The narrowest part of the femoral canal was 14 mm. A temporary screw was then inserted at the distal part of the bone graft. We then pulled the bone graft to the appropriate position in an antegrade manner by applying force with a hammer at the temporary screw and finally removed the temporary screw (Figure 2). The procedure was completed without reaming. The fracture site was further internally fixed with a Synthes variable angle locking distal femur plate (Synthes, Paoli, PA, USA) on the lateral surface of the femur (Figure 3). The operation time was 2 hours, and the amount of blood lost was 500 mL. The overall costs of the implants and the fibula bone graft were New Taiwan dollars (NTD) 95000. 

**Figure 4:**
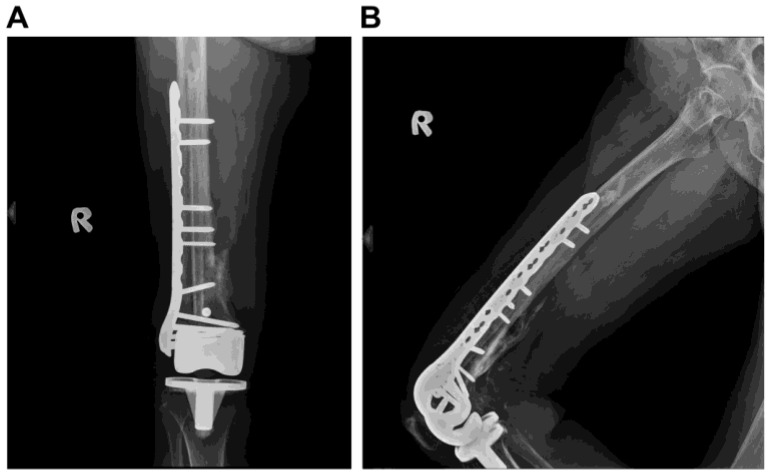
Radiographic images taken 4 months after the surgery. (**A**) Anteroposterior (AP) view of knee. (**B**) Lateral view of knee. The patient achieved a solid bone union.

**Figure 5:**
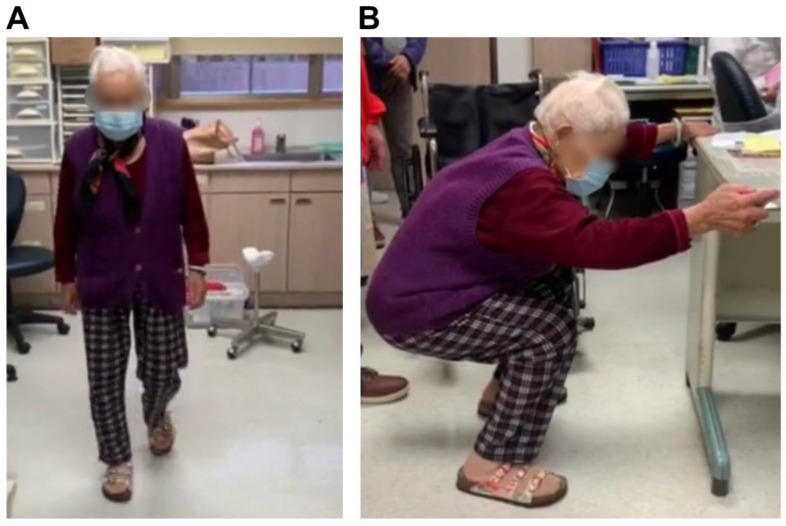
The patient can (**A**) walk without a walker and (**B**) squat while holding a table 4 months after the surgery.

Due to the relatively stable fixation technique, the patient was allowed immediate weight-bearing and early mobilization. She was discharged 5 days after surgery and underwent a regular clinical follow-up every month in the outpatient department. A walker was used for ambulation during the first 2 months. The fracture demonstrated union 4 months after the open reduction and internal fixation surgery, with only postoperative heterotopic ossification on the medial surface of the femur (Figure 4). Four months postoperatively, the patient could walk without a walker and squat while holding a table (Figure 5); she was satisfied with the outcome. No implant loosening nor varus/valgus deformity had occurred 2 years after surgery.

Written informed consent was obtained for the participation in and publication of this study. This study was approved by the institutional review board of the Shin-Kong Wu Ho-Su Memorial Hospital, Taiwan (Approval Number: 20210401R). It was conducted in accordance with the principles of the Declaration of Helsinki and CARE guidelines (https://www.equator-network.org).

## 3. Discussion

The aim of periprosthetic femur fracture treatment is to achieve a painless and stable knee joint and to maintain acceptable alignment [9,12]. Management of periprosthetic femur fractures includes conservative and surgical treatments [19]. A few studies have reported that conservative treatment can achieve good results [20,21]. Nevertheless, this treatment might lead to failure of bone health maintenance, a prolonged period of immobilization, reduced knee function, malunion, and nonunion [22]. Thus, the poor outcomes make conservative treatment for periprosthetic femur fractures less appealing [23,24]. 

Consequently, open reduction and internal fixation with a lateral locking plate and retrograde intramedullary nail (IMN) is recommended by surgeons to treat femoral periprosthetic fractures [25]. Although locking screw technology has improved the treatment of osteoporotic distal femur fractures, a relatively high nonunion rate with or without subsequent plate failure persists [12]. Nonunion rates obtained using retrograde IMN are similar to those obtained using locked plating; in such cases, excess fracture motion is not ideal for osteoporotic metaphyseal healing [26,27,28].

The use of either a laterally based locking plate or an IMN alone often warrants a protected weight-bearing status, instead of immediate weight-bearing, especially in osteoporosis [29,30,31]. However, patients undergoing surgical treatment with the combination of IMN and plate fixation are the exception [32]. The rationale behind this implementation lies in the assumption that the energy is more evenly distributed between the bone and the implants when combining both IMN and plate fixation in the distal femur. Theoretically, the nail fixation will shift the neutral, weight-bearing axis more medially along the anatomical axis of the femur, with added stability provided by the laterally based locked plate [15].

Although the combination of nail and plate fixation techniques achieved solid fixation, a few shortcomings were observed. First, once we drill the bone for placing the screws, we must avoid drilling on the nail. Thus, the screw length will be extremely short: approximately 8–12-mm-long and one cortex thick. However, in our surgical method, we need not worry about encountering the nail while drilling. In addition, drilling through the fibula bone graft may provide additional strength for fixing the bone graft. 

Moreover, while using the combination of nailing and plate fixation, we must incise the wound to pass the retrograde IMN. However, we used the same wound for bone graft placement without needing to include another wound in our study. 

In some hospitals, the fibula bone graft is separated into three-to-four parts after harvesting, each of which is preserved individually. This was not the case where an allogenous fibula bone graft was used to treat a periprosthetic femur fracture [15]. However, in contrast to a previous study published by Liporace, we used a longer allogenous fibula bone graft (with a length of approximately 24 cm). The diameter of the fibula diaphysis was similar to that of the femoral canal, which played a role similar to that of the retrograde IMN. 

In clinical practice, the outcome is considered very good if <5° varus or valgus malalignment in the coronal plane and <10° malalignment in the sagittal plane are observed. Furthermore, once the patient achieves a 90° range of motion of the knee and the shortening in length is >2 cm, they are considered to have a good prognosis [11]. In our case, valgus or varus malalignment or shortening changes were not observed 4 months after the surgery. Hence, a good outcome was obtained using an intramedullary non-vascularized allogenous fibula bone graft to treat a periprosthetic femoral fracture. This novel surgical technique will provide additional stability for patients with osteoporosis and TKA periprosthetic fractures. In addition, it facilitates immediate weight-bearing and early mobilization, which leads to better postoperative knee function. 

In clinical practice, few surgeons have applied non-vascularized autologous fibular strut grafts for treating femoral periprosthetic fractures [32]. However, in this case, we could not use an autogenous fibula bone graft. We were unable to harvest a 24 cm long fibula bone graft autogenously; many associated complications of using this bone graft have been reported, including common peroneal nerve injury, nonunion, weakness of the extensor *hallucis longus*, ankle joint instability, and stress fracture [33]. 

Nevertheless, this technique has some clinical limitations. First, although society is becoming increasingly open, there are a limited number of bone donors. Furthermore, in hospitals without adequate storage and sterilization techniques and equipment for bone graft restoration [33,34,35], it may be difficult to apply this surgical technique. Additionally, for long fibula bone graft insertion, each fibula can be used for one patient only. Thus, the limited source and amount of fibula donations are the primary limitations of using this surgical technique.

## 4. Conclusions

We have reported a successful case of using an intramedullary non-vascularized allogenous fibula bone graft to treat a periprosthetic femoral fracture in a patient with osteoporosis, resulting in solid fixation. The fibula bone graft not only acts as a structure for fixation but also allows the patient to achieve early weight-bearing and return to the previous functional score.

## Data Availability

Not applicable.
